# Improved GMP-compliant multi-dose production and quality control of 6-[^18^F]fluoro-L-DOPA

**DOI:** 10.1186/s41181-016-0009-1

**Published:** 2016-04-04

**Authors:** G. Luurtsema, H. H. Boersma, M. Schepers, A. M. T. de Vries, B. Maas, R. Zijlma, E. F. J. de Vries, P. H. Elsinga

**Affiliations:** Department of Nuclear Medicine and Molecular Imaging, University of Groningen, University Medical Center Groningen, Hanzeplein 1, PO Box 30.001, 9700 RB Groningen, The Netherlands

**Keywords:** PET, Radionuclide production, Radiochemistry, Automation, Quality control

## Abstract

**Background:**

6-[^18^F]Fluoro-L-3,4-dihydroxyphenylalanine (FDOPA) is a frequently used radiopharmaceutical for detecting neuroendocrine and brain tumors and for the differential diagnosis of Parkinson’s disease. To meet the demand for FDOPA, a high-yield GMP-compliant production method is required. Therefore, this study aimed to improve the FDOPA production and quality control procedures to enable distribution of the radiopharmaceutical over distances.

FDOPA was prepared by electrophilic fluorination of the trimethylstannyl precursor with [^18^F]F_2_, produced from [^18^O]_2_ via the double-shoot approach, leading to FDOPA with higher specific activity as compared to FDOPA which was synthesized, using [^18^F]F_2_ produced from ^20^Ne, leading to FDOPA with a lower specific activity. The quality control of the product was performed using a validated UPLC system and compared with quality control with a conventional HPLC system. Impurities were identified using UPLC-MS.

**Results:**

The [^18^O]_2_ double-shoot radionuclide production method yielded significantly more [^18^F]F_2_ with less carrier F_2_ than the conventional method starting from ^20^Ne. After adjustment of radiolabeling parameters substantially higher amounts of FDOPA with higher specific activity could be obtained. Quality control by UPLC was much faster and detected more side-products than HPLC. UPLC-MS showed that the most important side-product was FDOPA-quinone, rather than 6-hydroxydopa as suggested by the European Pharmacopoeia.

**Conclusion:**

The production and quality control of FDOPA were significantly improved by introducing the [^18^O]_2_ double-shoot radionuclide production method, and product analysis by UPLC, respectively. As a result, FDOPA is now routinely available for clinical practice and for distribution over distances.

## Background

Positron emission tomography (PET) with the radiopharmaceutical 6-[^18^F]fluoro-L-3,4-dihydroxyphenylalanine (FDOPA) is frequently used for measuring dopamine metabolism in vivo. Clinical applications of FDOPA PET are the detection and staging of neuroendocrine and brain tumors, and the differential diagnosis between Parkinson’s disease and other degenerative disorders of the central nervous system (Jager et al. 2008; Santhanam and Taïeb 2014). Since clinical indications for FDOPA PET imaging are increasingly being established, there will be a high demand for this radiopharmaceutical. Because of the growing clinical interest, there is a need for a high-yield GMP-compliant production method for FDOPA that enables the distribution of this radiopharmaceutical to other hospitals.

So far, the most common synthesis route to produce FDOPA is via an electrophilic substitution reaction with [^18^F]fluorine gas ([^18^F]F_2_). This approach has already been used for more than one decade (De Vries et al. 1999). A disadvantage of this synthesis route is the complex production of the [^18^F]F_2_ labeling reagent, giving low yields and requires the use of carrier fluorine gas. As a consequence of the use of [^18^F]F_2_, the radiochemical yield of the radiopharmaceutical is maximally 50 % and only a product with a low specific activity can be produced. The consequence of using FDOPA with low specific activity is that it could lead to adverse reactions such as flushes (Koopmans et al. 2006). To increase the specific activity and the relatively low radiochemical yield of FDOPA, two general approaches have been pursued: first, to develop a radiolabeling method for FDOPA using nucleophilic [^18^F]fluoride (Kuik et al. 2014), and second, to improve the [^18^F]F_2_ radionuclide production (Bishop et al. 1996; Bergman and Solin 1997) and the electrophilic labeling method. Considerable efforts were invested in the development of new synthesis approaches based on non-carrier-added [^18^F]fluoride as the starting material (Shen et al. 2009). These approaches generally required complex multistep synthetic strategies, resulting in low overall radiochemical yields. At this moment, new strategies are under development to obtain higher radiochemical yields (Lemaire et al. 2015; Zlatopolskiy et al. 2015).

In this article, we report on our efforts to optimize the FDOPA production using an improved method for the production of [^18^F]F_2_ for the electrophilic synthesis of FDOPA. The installation of a Cyclone 18 twin cyclotron (IBA, Belgium) recently allowed us to change our [^18^F]F_2_ production method from the ^20^Ne(d,α)^18^F single-shoot method (De Vries et al. 1999) to the ^18^O(p,n)^18^F double-shoot method (Bishop et al. 1996). As the Cyclone 18 twin cyclotron has a newly designed small volume target, the double-shoot production method needs less carrier F_2_ gas, which increases the specific activity of [^18^F]F_2_ in comparison to the single-shoot method using an MC17 Scanditronix cyclotron.

Finally, the overall aim of this study is to improve the radionuclide production and the electrophilic labeling method for FDOPA via [^18^O]_2_ double shoot approach using Cyclone 18 twin cyclotron. This improved method, leads to FDOPA with higher specific activity (FDOPA-H). The percentage radiochemical yield (%) and the amount of [^18^F]FDOPA radioactivity (practical yield), the radiochemical purity, specific activity is compared with conventional approach using MC17 Scanditronix cyclotron, which leads to FDOPA with a lower specific activity (FDOPA-L). Furthermore, an improved, more sensitive and accurate QC analysis for FDOPA is developed and validated using UPLC.

## Methods

### Materials for synthesis and analysis

For preparation of FDOPA the 6-trimethylstannyl-L-DOPA precursor was purchased from ABX (Germany). Dry chloroform >99 % for spectroscopy stabilized with amylene (stored on molecular sieves) and ammonium dihydrogen phosphate p.a. were obtained from Acros (Belgium) and 47 % hydrobromic acid, 25 % ammonia and diammonium hydrogenphosphate p.a. from Merck (Germany). A sterile solution for injection of ascorbic acid (100 mg/ml) was purchased from Centrapharm (Netherlands). A sterile 0.1 M sodium acetate solution, pH 4.7, was prepared in the hospital pharmacy. Reference compounds for calibration curves of FDOPA, L-DOPA and 6-hydroxy-DL-DOPA were purchased from respectively ABX (Germany) and Sigma Aldrich (Netherlands), HPLC supra gradient acetonitrile, used as solvent for LC-MS analysis, was obtained from Biosolve (Netherlands), ~98 % formic acid was bought from Fluka (Netherlands) and LC-MS grade water (>18.2 MΩ) was purchased from the Department of Clinical pharmacy & Pharmacology, UMCG. The reference compound leucine encephalin for calibration of the MS detector was purchased from Merck (Germany). For calibration of the MS, a solution was prepared of 200 μL 10 % formic acid, 100 μL 0.1 M sodium hydroxide (Merck, Germany) and 20 ml 80 % acetonitrile in water.

### The production of [^18^F]F_2_ using deuterons and proton particles

Deuteron particles were accelerated using a MC17 cyclotron. Carrier-added [^18^F]F_2_ was produced via the ^20^Ne(d,α)^18^F nuclear reaction. A 350-ml nickel target was filled with 0.25 % F_2_ (150 μmol) in neon and irradiated with 30 μA 8.5 MeV deuterons for 2 h. After irradiating the F_2_ target, [^18^F]F_2_ gas was transported via ~20 m ^1^/_8_ in. stainless steel tubing from Scanditronix MC-17 cyclotron to production laboratory. Passivation of transport lines was required.

Proton particles were accelerated using Cyclone 18 twin cyclotron. Production of [^18^F]F_2_ via the ^18^O(p,n)^18^F nuclear reaction was performed. A 35-ml aluminum target was filled with ^18^O-enriched oxygen gas ([^18^O]_2,_ > 97 %) at a pressure of 20 bar and irradiated with 35 μA 18 MeV protons for 1 h. The produced radioactivity is recovered from the target wall via a double shoot method. Therefore, the target was emptied to recover the [^18^O]_2_ gas using a cryogenic system and a vacuum was created inside the target to remove traces of [^18^O]oxygen. The target was filled with 24 bar 0.5 % F_2_ (75 μmol) in neon and irradiated for another 10 min. Passivation of transport lines was not necessary.

Transport of [^18^F]F_2_ gas from the Cyclone 18 twin cyclotron to the GMP laboratory is through 25 m long 1/8 in. stainless steel tubing with a flow of 250 ml.min^−1^.

### Synthesis of FDOPA

**Fig. 1:**
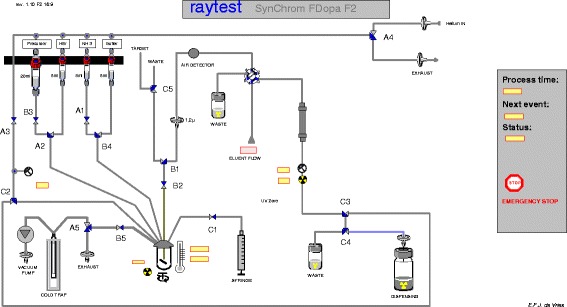
A schematic overview of FDOPA synthesis module including HPLC purification, collection of the end product and filter integrity test

**Fig. 2:**
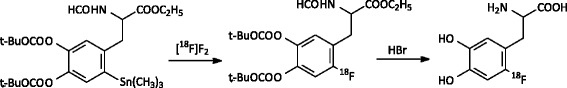
Synthesis of ^18^F-FDOPA

The synthesis procedure using [^18^F]F_2_ produced via the ^20^Ne(d,α)^18^F nuclear reaction was based on previous described synthesis (De Vries et al. 1999), but with some improvements and modifications. The modifications were; the activity was trapped into the reactor vial in the synthesis module (Raytest, Synchrom FDOPA F2, see schematic overview in Fig. 1) which was filled with 55–65 mg 6-trimethylstannyl-L-DOPA precursor in 3 ml of chloroform and cooled at a temperature of -20 °C. After trapping the [^18^F]F_2_ gas in the reaction mixture, the chloroform was evaporated at 70 °C. Thereafter, the hydrolysis using 2 ml 47 % hydrobromic acid was performed in 5 min at 130 °C. After hydrolysis, the reactor was cooled to 40 °C and 1.3 ml 25 % aqueous ammonia was added, followed by 1.3 ml buffer solution consisting of 1 M ammonium dihydrogen phosphate and 1 M diammonium hydrogenphosphate to neutralize the solution prior HPLC purification. A schematic overview of the synthesis is given in Fig. 1 and a schematic overview of the synthesis is illustrated in Fig. 2.

The product was eluted with a flow of 3 ml/min from two consecutive (connected in series) preparative HPLC columns (Hamilton PRP1 polymeric column in series (250 × 10 mm, 10 μm), Switzerland) with sterile 0.1 M sodium acetate, pH 4.7 and collected in a sterile vial with 100 μL ascorbic acid solution (100 mg/ml) to prevent oxidation. Aseptic filtration of the collected product was done via Millex LG filter and divided in three vials, patient batch, QC sample and a retain sample. Filtration and dispensing was performed in a hot cell with class A classification. The total synthesis time was 50–60 min.

One of the release criteria is the integrity of the used filter. To reduce the finger dose of the technicians we implemented an online pressure hold test directly in the module.

The synthesis procedure using [^18^F]F_2_ produced via the ^18^O(p,n)^18^F nuclear reaction was performed like described above, but with a slight modification. Due to low amount of carrier F_2_ gas, the amount of stannyl precursor was decreased to 30 mg (48.5 μmol).

### Quality control of FDOPA and identification of side products

For the determination of the specific activity, the radiochemical and chemical purity of FDOPA an QC analysis was performed on conventional HPLC system. To improve the analysis with respect to retention times and resolution we implemented an Acquity Ultra Performance Liquid Chromatography (UPLC®) with UV and an online radioactivity detector from Berthold (Flowstar LB513) in our laboratory. For a direct comparison on retention times of L-DOPA and FDOPA and the resolution of the separation, same samples were injected on the two analytical methods. The conventional HPLC method was performed on Waters system (pump model 515 and tunable UV detector model 486), using a Hamilton PRP-1 150 × 4,1 mm column, with a eluent of sodium citrate 8,71 g and sodium dodecylsulfon 0,8 g in 900 ml H2O pH 2,9/100 ml acetonitrile and a flow rate of 1 ml/min.

A UPLC method was executed, using a Waters ACQUITY UPLC® HSS T3 1.8 μm, 3.0 × 50 mm analytical column with an eluent of 0.05 M phosphate buffer pH 2.5 with an isocratic flow of 0.8 ml/min. For both methods a wavelength of 254 nm was used for UV-detection. As specified in the European pharmacopeia 8, FDOPA, L-DOPA, 6-hydroxy-DOPA were used as reference standards. An additional test on the UPLC was performed to provide information about the stability of FDOPA during the time of transport (max 8 h). Over a period of 8 h at room temperature, samples were taken at different time points for analysis of the radiochemical purity using UPLC.

Identification of specific compounds like; FDOPA, L-DOPA, 6-hydroxy-DOPA was performed on a Waters (Milford, MA, USA) UPLC H-class system coupled to a Waters Xevo® G2 QTof mass spectrometer (UPLC-MS). Samples (1 μL) were injected onto a Waters 5.0 × 2.1 mm I.D., 1.7 μm Ethylene Bridged Hybrid (BEH) C_18_ column and eluted at a flow rate of 0.6 ml/min with a 1.5-min linear gradient of 10 mM ammonium bicarbonate pH 9.4 and acetonitrile, starting at 2 % and ending at 80 % acetonitrile.

Negative electrospray ionization in resolution mode was used. Both MS and MS^E^ scans were performed simultaneously in a mass range from 50 to 1200 Da. MS^E^ is a method of data acquisition that records exact mass precursor and fragment ion information from every detectable component in a sample. The following settings were used: capillary voltage 0.5 kV, sampling cone 65 V, extraction cone 4.0 V, source temperature 150 °C and desolvation temperature 500 °C. The collision energy was set to 6 V during the MS acquisition and it was ramped from 20 to 45 during the MS^E^ acquisition. Leucine-Enkephalin was used as a lock mass (m/z 554.2615 Da → negative mode) with a concentration of 2 ng/μL, at a flow rate of 20 μL/min. The capillary voltage was set to 2.5 kV.

## Results

### [^18^F]F_2_ production

The radioactivity amount of [^18^F]F_2_ using irradiation with deuterons ranged between 4 and 8 GBq (70-100 MBq. μAh^−1^) and using proton irradiation, the [^18^F]F_2_ production ranged between 25 and 35 GBq (800-1100 MBq. μAh^−1^). Both activities were measured under the same conditions in the reaction vial of the module, 10 min after EOB. The total time, including irradiations, flushing, pre- and double shoot beam, was 150 min using deuteron and 90 min using the double shoot proton procedure.

### FDOPA synthesis

Results of the synthesis of FDOPA with [^18^F]F_2_ from both production methods are summarized in Table 1. The radiochemical yield (%) for the production of FDOPA-H was higher in comparison to the FDOPA-L-production. The radiochemical yield (%) was calculated from trapped activity in the reactor. The average overall amount of [^18^F]FDOPA and the average specific activity were measured at the end of synthesis (EOS). The average specific activity of FDOPA-H was > 15 times higher compared to FDOPA-L.

**Table 1:** An overview is given of the [^18^F]_2_ production method, number of productions, the radiochemical yield, the specific activity, radiochemical yield, radiochemical purity and the amount of FDOPA activity

	Method [^18^F]_2_ production	Number of productions (n)	Radiochemical yield (%)	Specific activity (GBq/mmol)	Radio-chemical Purity (%)	Amount of FDOPA activity (MBq)
FDOPA-Lower	^20^Ne(d,α)^18^F single-shoot	68	15 ± 5	8.5 ± 3.3	97 ± 3	526 ± 192
FDOPA-Higher	^18^O(p,n)^18^F double-shoot	42	23 ± 4	121 ± 27	97 ± 3	4521 ± 967

### FDOPA quality control and identification of impurities

Concentrations of FDOPA and L-DOPA meet the pharmacopeia specifications (FDOPA 15 mg/dose and L-DOPA 1 mg/dose, respectively) and were used to calculate the SA which is a pre-release criterion (Table 2).

**Table 2:** Concentrations and identification of FDOPA and L-DOPA (g/l) and side products in FDOPA-L and FDOPA-H batches analyzed with UPLC-MS (both *n* = 6)

FDOPA-L				
MC17- cyclotron	FDOPA 214.052 Da (g/L)	L-DOPA 196.061 Da (g/L)	6-hydroxy-DL-DOPA 212.056 Da	FDOPA-quinone 212.036 Da
X ± SD	1.21 ± 0.15	0.04 ± 0.04	-	+
FDOPA-H				
Cyclone 18 twin cyclotron	FDOPA 214.052 Da (g/L)	L-DOPA 196.061 Da (g/L)	6-hydroxy-DL-DOPA 212.056 Da	FDOPA- quinone 212.036 Da
X ± SD	0.53 ± 0.10	0.007 ± 0.003	-	-

+ = side product detected and – means not detectable

The total analysis time using UPLC was 3 min, whereas execution of the HPLC-method took 15 min. The retention times of FDOPA and L-DOPA measured with UPLC were 1.5 and 1.1 min, respectively. The retention time on HPLC for FDOPA was 7 min and 5 min for L-DOPA. The calculated resolution factor (Rf) was 3.0 and 1.4 for UPLC and HPLC, respectively.

**Fig. 3:**
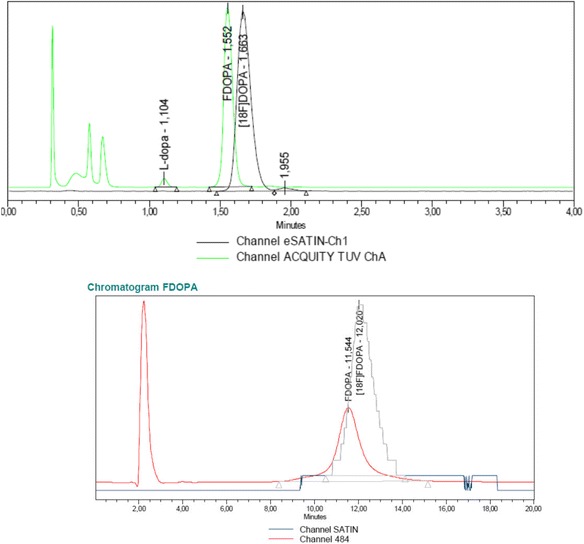
An example of a typical chromatogram of FDOPA -L performed with UPLC-UV and radioactivity detector (*upper*). *Green* is UV and the *black line* is RA signal. Notice that because the different detector position there is a delay in RA-signal. *Below*, a typical presentation of a chromatogram of FDOPA -L performed with HPLC-UV and radioactivity detector

The radiochemical purity, the specific activity and the concentration FDOPA and L-DOPA were determined using the UPLC method and all met the pharmacopeia requirements. FDOPA was at least stable (>95 % RCP) during 8 h after synthesis. An example of QC chromatogram performed with UPLC and HPLC coupled with UV and radioactivity detector is given in Fig. 3.

**Fig. 4:**
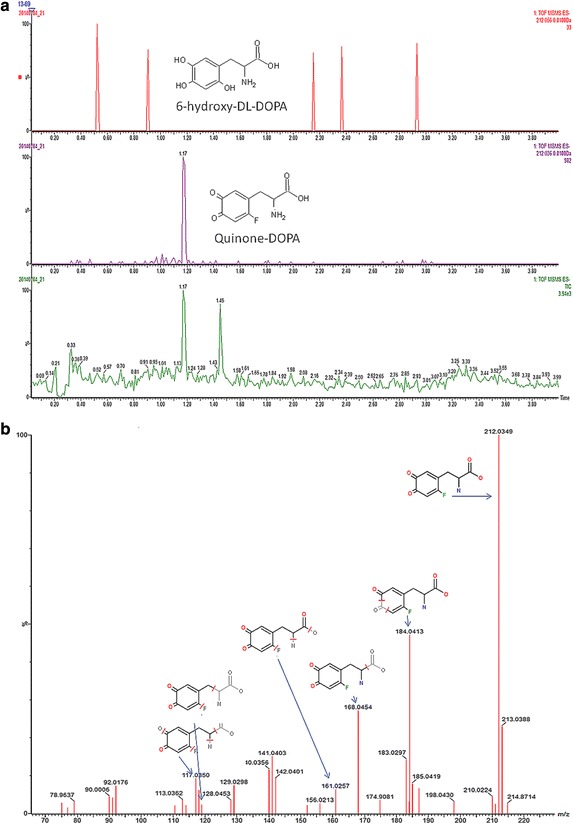
**a** Mass spectra of low specific activity FDOPA sample. No 6-hydroxy-DOPA was found in the sample (*above*). Peak of FDOPA-quinone (*middle*) was detected and the total ion current chromatogram is presented *below*. **b** Proposed FDOPA- quinone fragments

Additionally, both FDOPA and L-DOPA were identified using exact mass (*n* = 6 for FDOPA-H, *n* = 6 FDOPA-L). However, identification of 6-hydroxy-DOPA in the drug product was not possible. In contrast, a molecule with almost the same mass as 6-hydroxy-DOPA (212.036 Da), but with a different retention time as the reference standard was found. Calculation of the elemental composition from the exact mass and fragment ion information using MS^E^ scans (Fig. 4b) proved that this product is 2-amino-3-(6-fluoro-3,4-dioxocyclohexa-1,5-dienyl)propanoic acid (FDOPA-quinone), which can be formed by oxidation of FDOPA. FDOPA–quinone was only detected in lower specific activity FDOPA samples (Fig. 4a). We did not find changes in FDOPA-quinone concentration over time. Because no reference compound FDOPA-quinone was available, it was not possible to quantify FDOPA-quinone concentrations in the samples (see Fig. 4a and b for the mass spectra).

## Discussion

This study demonstrates that optimized radionuclide production, via a proton double-shoot method on [^18^O]_2_ leads to significant higher amounts of FDOPA with higher specific activity.

Although the ratio μmol precursor versus carrier F_2_ was equal for both syntheses, the radiochemical yield with the improved FDOPA-H method was higher compared to the conventional FDOPA-L. This phenomenon could be due to the higher purity of [^18^F]F_2_. Intensive cleaning of the target using flushing and vacuum was always done after the first shoot and therefore the [^18^F]F_2_ produced via the double-shoot method may contain less chemical impurities and therefore its reaction with the [^18^F]FDOPA precursor appears to be more efficient. Taken together, it is evident that the new method leads to significantly higher radiochemical yields and together with higher radionuclide production yield of [^18^F]F_2_ with less carrier F_2_ consequently more amount of FDOPA with higher specific activity was achieved.

As a result of the higher specific activity, lower amounts of carrier FDOPA need to be administered to the patient, which reduces the risk on side effects, such as carcinoid crisis (Koopmans et al. 2006).

In general, it can be concluded that, although the complex radionuclide production of [^18^F]F_2_ gas, the radiochemistry via electrophilic substitution is quite straight forward resulting in short reaction times. Disadvantages of using [^18^F]F_2_ gas, as labeling reagent, is the theoretical maximum radiochemical yield of 50 % and the corrosive character of [^18^F]F_2_ gas which can lead to malfunctioning of valves and equipment.

During this study, 6-hydroxy-DOPA could not be detected in the produced FDOPA batches, irrespective of its specific activity. Another impurity, the so called FDOPA-quinone, could be detected, but only in FDOPA batches with lower specific activity. The identification of this impurity was based on the exact mass, as determined by UPLC-MS, in combination with the proposed fragmentation pattern for this product. The difference in the exact mass of 6-hydroxy-DOPA and FDOPA-quinone is only 0.02 Da, but could be readily detected by UPLC-MS. Together with the observed fragments, FDOPA-quinone could be reliably identified with this method. Why FDOPA-quinone was only identified in the FDOPA-L batch is not completely clear. The major difference in both production methods are the used concentrations of precursor, radionuclide production of [^18^F]F_2_ and the differences in SA of FDOPA.

Performing state-of-the-art quality control analysis using UPLC in combination with radioactivity detector and MS-TOF leads to accurate identification of the impurities. Using UPLC-MS, impurities, like the FDOPA-quinone, in low molecular concentrations can be identified. This can provide more accurate information which is needed for the release of radiopharmaceuticals. In this study, we have shown that 6-hydroxy-DOPA is not formed as an impurity in FDOPA batches. There is no logical chemical pathway that could lead to the formation of this impurity, as hydrolysis of the trimethylstannyl precursor should lead to the formation of L-DOPA (and trimethyltin hydroxide). Considering the similarity in molecular weight, it seems plausible that FDOPA-quinone has been falsely identified as 6-hydroxy-DOPA in the past. Therefore, an adjustment of the FDOPA monograph in the European Pharmacopeia should be considered.

It is expected that the population of patients with endocrine tumors will grow in the coming years and the application of FDOPA will grow for differential diagnosis between Parkinson’s disease and other degenerative disorders. In order to meet with the increasing demand for FDOPA PET, we improved the [^18^F]F_2_-based electrophilic production method of FDOPA to make it suitable for production of FDOPA for multiple patients in our institute and distribution over distance. The fact that the described FDOPA production is GMP compliant, our institute was granted an exemption to have a marketing authorization by the government to distribute FDOPA to other hospitals without a dossier for marketing authorization. This exemption is applicable for the Netherlands, because FDOPA is a licensed radiopharmaceutical, but not available for use, since the current manufacturing authorization holder does not produce FDOPA. With the described production method, production of FDOPA batches for distribution to other hospitals became feasible.

## Conclusion

The production and quality control of FDOPA were significantly improved by introducing the [^18^O]_2_ double-shoot radionuclide production method, improved synthesis, and product analysis by UPLC, respectively. As a result, FDOPA is now routinely available for clinical practice and for distribution over distances. Adjustment of the FDOPA monograph in the European Pharmacopeia should be considered.
